# Flame-like Calcifications in Werner Syndrome

**DOI:** 10.1210/jcemcr/luad099

**Published:** 2023-08-24

**Authors:** Yasutaka Tsujimoto, Hironori Bando

**Affiliations:** Division of Diabetes and Endocrinology, Department of Internal Medicine, Kobe University Hospital, Kobe 650-0017, Japan; Division of Diabetes and Endocrinology, Department of Internal Medicine, Kobe University Hospital, Kobe 650-0017, Japan

**Keywords:** diabetes mellitus, progeroid syndrome, foot disease

## Image Legend

**Figure 1.**
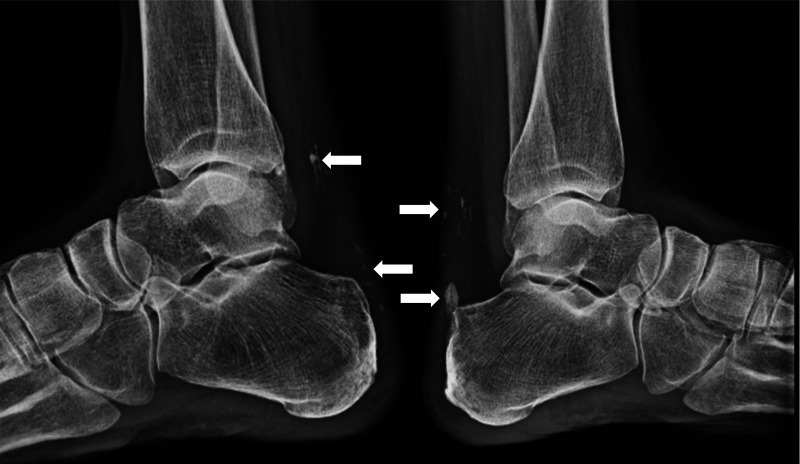
White arrows show calcifications in the Achilles tendons.

A 42-year-old Japanese woman was referred for diabetes mellitus with onset at age 39. Her medical history included nonalcoholic fatty liver disease, dyslipidemia, and primary hypogonadism. Her height, body weight, and body mass index were 159.5 cm, 43.7 kg, and 17.1 kg/m^2^, respectively. Fasting plasma glucose was 5.27 mmol/L (95 mg/dL) and serum insulin was 156.42 pmol/L (21.8 μU/mL), suggesting high insulin resistance. She had a hoarse voice, thinning hair, and scleroderma-like skin changes, matching progeria. She complained of heel pain and skin ulceration on her feet. Her sister and brother had a similar medical history and physical findings. Foot radiography showed flame-like calcifications of the Achilles tendon (Fig. 1), which were frequently shown in Werner syndrome [1]. Genetic testing revealed a compound heterozygous variant of *WRN* gene (c.3139-1G > C and c.3383 + 1G > T), resulting in a diagnosis of Werner syndrome. Werner syndrome is an autosomal recessive disorder and progeroid syndrome. A patient with Werner syndrome often has comorbidities such as diabetes mellitus, foot disease, arteriosclerosis, and malignancies, which worsen the prognosis. The hallmark of glucose intolerance in patients with Werner syndrome is high insulin resistance without obesity [2]. Werner syndrome could be a cause of young adult–onset diabetes mellitus and its clue of diagnosis may be at the feet.
